# Detergent-Induced Stabilization and Improved 3D Map of the Human Heteromeric Amino Acid Transporter 4F2hc-LAT2

**DOI:** 10.1371/journal.pone.0109882

**Published:** 2014-10-09

**Authors:** Marcel Meury, Meritxell Costa, Daniel Harder, Mirko Stauffer, Jean-Marc Jeckelmann, Béla Brühlmann, Albert Rosell, Hüseyin Ilgü, Karin Kovar, Manuel Palacín, Dimitrios Fotiadis

**Affiliations:** 1 Institute of Biochemistry and Molecular Medicine, and Swiss National Centre of Competence in Research (NCCR) TransCure, University of Bern, Bern, Switzerland; 2 Institute of Biotechnology, School of Life Sciences and Facility Management Zürich University of Applied Sciences (ZHAW), Campus Grüntal, Wädenswil, Switzerland; 3 Institute for Research in Biomedicine (IRB Barcelona), Barcelona, Spain; 4 Centro de Investigación Biomédica en Red de Enfermedades Raras, Barcelona, Spain; 5 Department of Biochemistry and Molecular Biology, Faculty of Biology, University of Barcelona, Barcelona, Spain; Rosalind Franklin University, United States of America

## Abstract

Human heteromeric amino acid transporters (HATs) are membrane protein complexes that facilitate the transport of specific amino acids across cell membranes. Loss of function or overexpression of these transporters is implicated in several human diseases such as renal aminoacidurias and cancer. HATs are composed of two subunits, a heavy and a light subunit, that are covalently connected by a disulphide bridge. Light subunits catalyse amino acid transport and consist of twelve transmembrane α-helix domains. Heavy subunits are type II membrane N-glycoproteins with a large extracellular domain and are involved in the trafficking of the complex to the plasma membrane. Structural information on HATs is scarce because of the difficulty in heterologous overexpression. Recently, we had a major breakthrough with the overexpression of a recombinant HAT, 4F2hc-LAT2, in the methylotrophic yeast *Pichia pastoris*. Microgram amounts of purified protein made possible the reconstruction of the first 3D map of a human HAT by negative-stain transmission electron microscopy. Here we report the important stabilization of purified human 4F2hc-LAT2 using a combination of two detergents, i.e., n-dodecyl-*β*-D-maltopyranoside and lauryl maltose neopentyl glycol, and cholesteryl hemisuccinate. The superior quality and stability of purified 4F2hc-LAT2 allowed the measurement of substrate binding by scintillation proximity assay. In addition, an improved 3D map of this HAT could be obtained. The detergent-induced stabilization of the purified human 4F2hc-LAT2 complex presented here paves the way towards its crystallization and structure determination at high-resolution, and thus the elucidation of the working mechanism of this important protein complex at the molecular level.

## Introduction

Human heteromeric amino acid transporters (HATs) are antiporters composed of two subunits, a heavy (SLC3 family) and a light subunit (SLC7 family) covalently linked by two conserved cysteine residues [1]–[3]. Light subunit members are also called L-type amino acid transporters (LATs) and belong to the large amino acids, polyamines and organocations (APC) transporter superfamily [4]. The light subunit constitutes the transport system for large and neutral amino acids, whereas the heavy subunit is required for the functional expression of the HAT in plasma membranes [5]–[7]. Exchange of amino acids by HATs across membranes is driven in a Na^+^-independent manner with a 1∶1 stoichiometry [8], [9]. In humans, two heavy subunits have been identified, i.e., rBAT and 4F2hc [1]. The latter is involved in the formation of heterodimeric complexes with six different light subunits, i.e., LAT1, LAT2, y^+^LAT1, y^+^LAT2, asc1 and xCT [3]. Due to the widespread expression of these transport systems in organs and tissues, HATs are determining factors for a variety of human diseases, e.g., aminoacidurias (cystinuria and lysinuric protein intolerance) [7], [10], [11], tumor cell growth [12], [13], Kaposi's sarcoma-associated herpesvirus infection [14], [15] and cocaine relapse [16].

Despite the relevance of HATs in human physiology, detailed structural information has only been published for the extracellular domain of human 4F2hc [17]. The SLC3 family member 4F2hc is a type II membrane N-glycoprotein. 4F2hc is composed of a single transmembrane (TM) α-helix, an intracellular N-terminus and a large extracellular C-terminus [1]. X-ray crystallography revealed that the structure of the 4F2hc-ectodomain (ED) is similar to bacterial glucosidases with the typical triose phosphate isomerase (TIM) barrel (βα)_8_ fold and eight additional antiparallel β-strands [17]. In contrast to the bacterial homologs, human 4F2hc is missing the catalytic key residues for glucosidase activity [17]. On the structure of LATs, cysteine-scanning mutagenesis studies of xCT identified a twelve TM α-helix topology with intracellular N- and C-termini [18]. Recently, with the crystal structures of AdiC (L-arginine/agmatine antiporter) [19]–[22], ApcT (broad-specificity amino acid transporter) [23] and GadC (glutamate/GABA antiporter) [24], atomic models of prokaryotic APC transporters have become available. These models provide insights into the architecture and molecular transport mechanisms of APC superfamily members. LATs are likely to share a common architecture with their distantly related (≤20% amino acid sequence identity) prokaryotic APC homologs, but to date little information is available on the structure of HATs.

In a recent publication on the expression screening of human HATs in the methylotrophic yeast *Pichia pastoris* (*P. pastoris*), we identified 4F2hc-LAT2 as a promising candidate for functional and structural studies of HATs [25]. Furthermore, a first glimpse on the supramolecular organization of HATs could be obtained recently for 4F2hc-LAT2 by transmission electron microscopy (TEM), single particle analysis, cysteine scanning mutagenesis and disulphide mapping [26]. Using these experimental data, *in silico* docking analyses were performed with the 4F2hc-ED structure and a homology model of LAT2 to predict the contact sites at the heterodimer interface. Interactions were found between 4F2hc and the external loop regions of LAT2 indicating that the heavy subunit acts as a scaffold protein for the stability of the transporter complex [26]. Stabilization of LAT2 by the 4F2hc-ED was further corroborated by biochemical experiments. In these two studies [25], [26], His-tagged 4F2hc and StrepII-tagged LAT2 were overexpressed in *P. pastoris* and the heterodimer purified from n-dodecyl-*β*-D-maltoside (DDM) solubilized membranes. However, purification in DDM resulted not only in 4F2hc-LAT2 heterodimers, but also in 4F2hc and LAT2 monomers (from disrupted heterodimers) and aggregates [25], [26]. Size exclusion chromatography (SEC) profiles of DDM-purified 4F2hc-LAT2 indicated, in addition to the heterodimer peak, heterogeneous protein populations characterized by a prominent aggregates/oligomers peak [25]. Substrate binding to purified 4F2hc-LAT2 could not be determined due to the relatively low stability of the HAT in DDM [26]. Nevertheless, transport activity of the recombinant human 4F2hc-LAT2 complex was demonstrated by two different methods, i.e., uptake experiments using Pichia cells and proteoliposomes reconstituted from isolated, DDM-solubilized membranes.

## Results and Discussion

We significantly improved the stability of detergent-solubilized 4F2hc-LAT2 for ligand-binding experiments and structural characterization by scintillation proximity assay (SPA) and TEM/single particle analysis, respectively. The stabilization of 4F2hc-LAT2 was successfully achieved by membrane solubilisation and protein purification using the two detergents DDM and lauryl maltose neopentyl glycol (LMNG), and cholesteryl hemisuccinate (CHS) simultaneously. CHS [27], [28] and LMNG [29] were shown to be beneficial for stability and retaining the function of purified mammalian membrane proteins (see indicated references for details on possible stabilizing mechanisms of CHS and LMNG). Inspired by these reports, we modified our original purification protocol [25] and included LMNG and CHS in addition to DDM.

**Figure 1:**
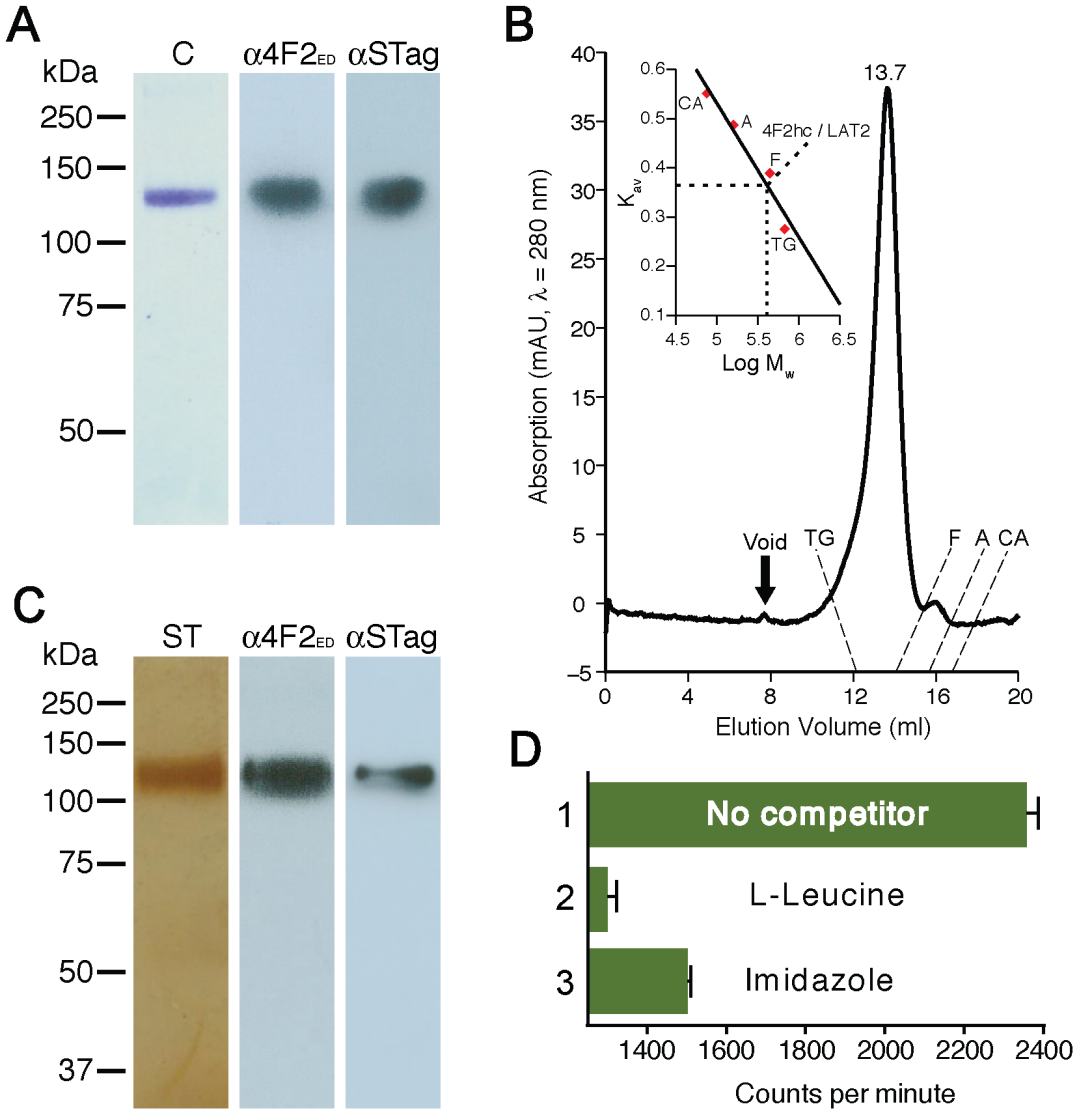
SDS-PAGE, Western blot analysis, SEC and SPA of 4F2hc-LAT2 purified in DDM, LMNG and CHS. (A) Coomassie Blue-stained 10% SDS/polyacrylamide gel (lane C) of 4F2hc-LAT2 after the two affinity chromatography steps. The complex runs as a prominent band at ∼125 kDa. Western blot analysis using anti-4F2hc (lane α4F2_ED_) and anti-StrepTagII (lane αSTag) antibodies indicated the presence of intact heterodimers only, i.e., no 4F2hc or LAT2 from disrupted complexes. (B) SEC of purified 4F2hc-LAT2 indicated a prominent almost symmetrical elution peak at 13.7 ml. (C) Silver-stained 10% SDS/polyacrylamide gel of purified 4F2hc-LAT2 after gel filtration (lane ST; from peak fraction). Again, one single band is visible corresponding to the heterodimer. Integrity of the complex was further supported by Western blotting using anti-4F2hc (lane α4F2_ED_) and anti-StrepTagII (lane αSTag) antibodies. (D) Radioligand-binding assay by SPA using purified 4F2hc-LAT2 and [^3^H]L-leucine. Bar 1: Binding of the radiolabelled substrate L-leucine to 4F2hc-LAT2, which is bound to scintillation beads, induces SPA signal. As expected, SPA signal was abolished by addition of 4 mM cold L-leucine (bar 2; competitive inhibition) or 100 mM imidazole (bar 3; detachment of the protein from the SPA beads). Bars represent mean ± SEM from triplicates. One representative of three similar independent experiments is shown.

**Figure 2:**
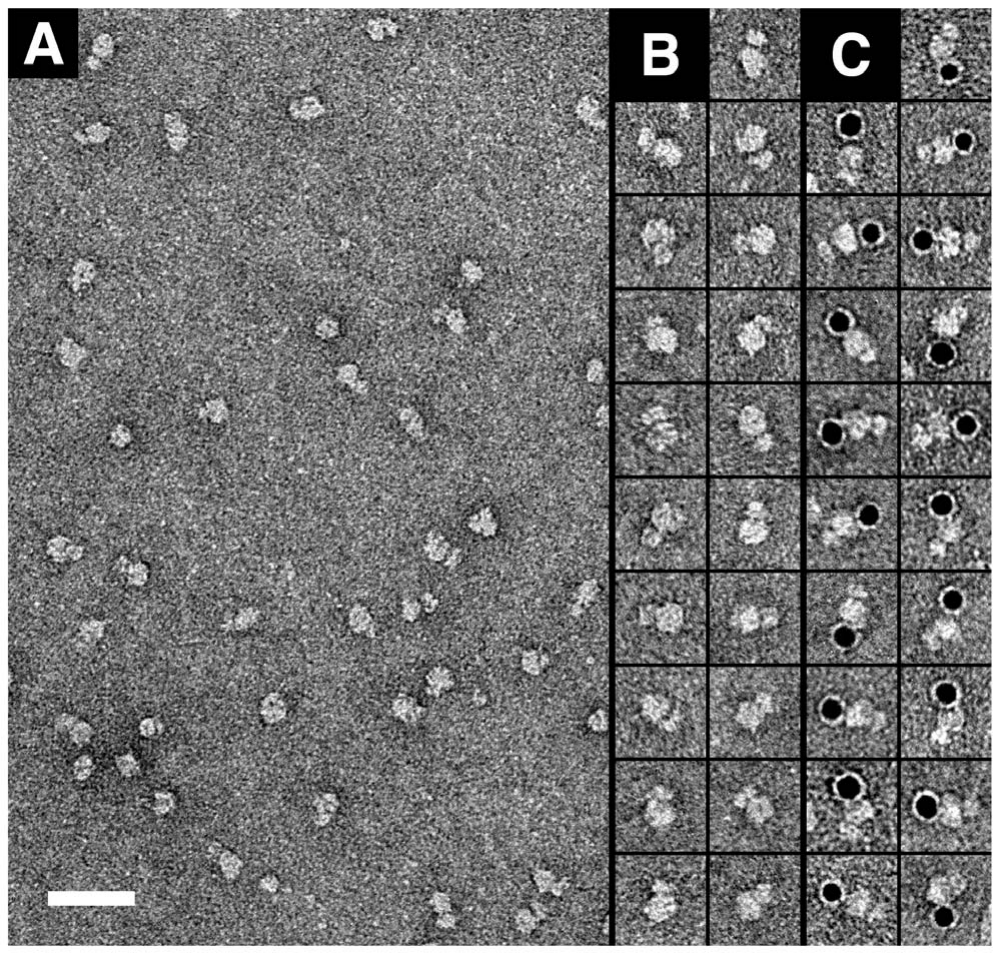
TEM and Nanogold labelling of purified human 4F2hc-LAT2 heterodimers. (A) Electron micrograph of purified negatively-stained 4F2hc-LAT2 heterodimers. Galleries of unlabelled (B) and 5 nm Ni-NTA-Nanogold labelled (C) 4F2hc-LAT2 heterodimers. Bilobed particles with two different sized domains are clearly visible. The 5 nm Ni-NTA Nanogold spheres are localized to the larger domains of the heterodimer. The scale bar in (A) represents 50 nm. The frame size of the boxes in (B) and (C) represent 28 nm.

We purified 4F2hc-LAT2 by sequential cobalt and Strep-Tactin affinity chromatographies [25] in the presence of DDM, LMNG and CHS. The integrity, homogeneity and stability of purified 4F2hc-LAT2 heterodimers was analysed by SDS-PAGE, Western blot analysis and SEC (Fig. 1). SDS-PAGE showed a single protein band with a molecular mass of ∼125 kDa (Fig. 1A; lane C) corresponding to the purified heterodimer. Importantly, no 4F2hc and LAT2 monomers, i.e., disrupted heterodimers, or aggregates/oligomers were detected by Western blotting using anti-4F2hc and anti-StrepTagII antibodies in stark contrast to purification with DDM (see Fig. 4A in Ref. [25] and Fig. S1 for comparison). The presence of LMNG and CHS in addition to DDM even prevented disruption of 4F2hc-LAT2 heterodimers six days after purification (Fig. S1). Only some unspecific formation of dimers and higher oligomers of heterodimers was observed after such periods of time (Fig. S1A, *lower*). In contrast, Western blot analysis of DDM-purified 4F2hc-LAT2 detected significant amounts of 4F2hc and LAT2 monomers from disrupted heterodimers and high amounts of aggregates after the same timespan (Fig. S1B, *lower*). SEC profiles of 4F2hc-LAT2 purified with DDM/LMNG/CHS indicated a single almost symmetric peak at a retention volume of 13.7 ml (Fig. 1B). By comparing retention volumes of purified 4F2hc-LAT2 and soluble molecular weight markers, the molecular mass of the complex was estimated to be ∼406 kDa indicating a significant amount of bound DDM, LMNG and CHS. Similar to the purified protein (Fig. 1A), SDS-PAGE and Western blot analysis of the SEC peak fraction demonstrated full integrity of the 4F2hc-LAT2 heterodimer (Fig. 1C). In summary, purification of 4F2hc-LAT2 in DDM, LMNG and CHS significantly increased the stability of the complex paving the way for new functional and structural studies. First, we performed substrate binding to purified heterodimer using the SPA as previously reported for transport proteins [30], [31]. Fig. 1D (bar 1) shows clear binding of the substrate [^3^H]L-leucine to purified 4F2hc-LAT2 by SPA. As expected, the SPA signal was lost by the addition of 4 mM L-leucine (Fig. 1D, bar 2) indicating competitive binding of unlabelled substrate to the heterodimer. The SPA signal was also lost by the addition of 100 mM imidazole (Fig. 1D, bar 3), which displaced the 4F2hc-LAT2 from the SPA beads. Second, the integrity, homogeneity, stability and ability to bind substate of DDM/LMNG/CHS-purified 4F2hc-LAT2, as determined biochemically, prompted us to perform TEM and single particle analysis. Fig. 2A shows a representative overview electron micrograph of negatively-stained 4F2hc-LAT2 complexes. In contrast to 4F2hc-LAT2 purified only in DDM [26], small protein aggregates, and 4F2hc or LAT2 monomers from disrupted heterodimers were rarely found supporting the high integrity, homogeneity and stability of the complex when purified in DDM/LMNG/CHS. As for DDM-purified 4F2hc-LAT2 [26], particles had a distinct shape composed of two globular domains of different sizes (Fig. 2B). This characteristic feature is even more prominent in calculated class averages (Fig. S2A) compared to the raw images. In Rosell *et al.* we assigned the large and small domains of DDM-purified heterodimer particles to LAT2 and 4F2hc, respectively [26]. To further support this assignment experimentally, we performed labelling of the N-terminally located His-tag of 4F2hc using 5 nm Ni-NTA-Nanogold probes. The N-terminus of 4F2hc is located on the cytoplasmic side followed by a single TM α-helix and the large C-terminal ED, which is located on the extracellular side. On electron micrographs, the Nanogold probe was identified at the large domain of 4F2hc-LAT2 heterodimers and opposite to the small domain (Fig. 2C) supporting our previous assignment [26]. We calculated a 3D reconstruction of DDM/LMNG/CHS-purified 4F2hc-LAT2 from 27'921 negatively-stained complexes (Fig. S2B). The resolution of the obtained 3D map was estimated to 20 Å (Fig. S2C). This resolution should be considered with caution because the 3D reconstruction was obtained from negatively-stained samples. The distribution of the particle orientations after the last refinement cycle is shown in Fig. S2D and indicates a homogenous angular distribution of single particle projections.

**Figure 3:**
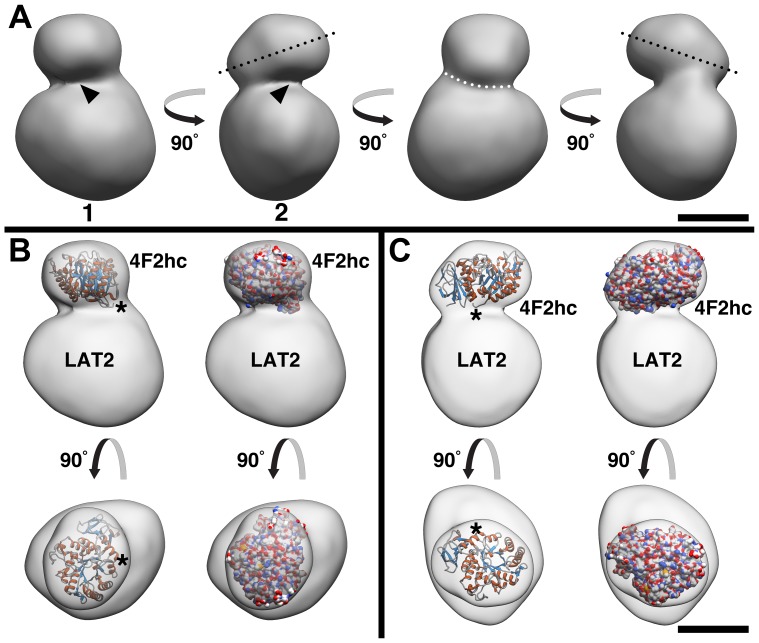
3D reconstruction of human 4F2hc-LAT2 purified in DDM, LMNG and CHS. (A) Side views of the 3D map calculated from 27'921 projections of negatively-stained heterodimer particles (initially 28'993 picked). 4F2hc-LAT2 has a bilobed structure and consists of a smaller tilted density located on top of a larger density (tilt indicated by black dotted lines). As a consequence of the tilt, the 3D map features a distinct cavity (arrowheads) and on the opposite side a seal (marked by a white, dotted curve). Based on the Fourier shell correlation curve (0.5 criterion) the resolution is 20 Å (Fig. S2C). (B) and (C) Different views of the 4F2hc-LAT2 3D map with the fitted crystal structure of the 4F2hc-ED (PDB ID: 2DH2). Asterisks in (B) and (C) indicate the location of the N-terminus in the 4F2hc-ED crystal structure. The 4F2hc-ED structure is represented as cartoon (α-helices and β-strands in red and blue, respectively) and surface (CPK colours) models. The scale bar represents 50 Å.

In congruence with the class averages, the 3D model of 4F2hc-LAT2 exhibits a particular shape consisting of two differently sized densities (Fig. 3A; for additional views see Fig. S2B). The overall architecture of the 3D model calculated from DDM/LMNG/CHS-stabilized 4F2hc-LAT2 is similar to the 3D reconstruction we previously published [26]. Both models feature a smaller density on top of a larger one, with the former being clearly tilted (Fig. 3A, black dotted lines). As a consequence of the tilt, the 3D map possesses a cavity (Fig. 3A, arrowheads) and a seal (Fig. 3A, white dotted curve) in the interface region between the smaller and larger density. We fitted the X-ray structure of 4F2hc-ED [17] into the smaller density of our 3D model. This resulted in a similar orientation of the ED structure as seen in our previous 3D map calculated from 4F2hc-LAT2 purified in DDM (Fig. 3B and 3C) [26]. The orientation of the ED reveals the approximate localization of the N-terminal TM α-helix of the heavy-chain 4F2hc (Fig. 3B and 3C, indicated by asterisks). The previous 3D reconstruction of 4F2hc-LAT2 purified in DDM was calculated from 15,210 single particle projections. In contrast, the 4F2hc-LAT2 3D map presented here was calculated from 27'921 negatively-stained complexes, thus containing almost twice the number of projections. More, importantly, the high protein quality of isolated 4F2hc-LAT2 when purifying with the DDM, LMNG and CHS combination allowed us to collect a significantly higher number of projections of intact complexes. We compared the 3D maps of 4F2hc-LAT2 calculated from protein purified in DDM [26] and DDM/LMNG/CHS (Fig. S3). While the densities of the EDs were similar, differences were found in the densities corresponding to LAT2. In the ellipsoid shaped LAT2 densities, the short axes differed by 14 Å (85 Å minus 71 Å; Fig. S3A) while the long axes were identical (i.e., 99 Å; Fig. S3B). Because of the same long axes, we attribute the obtained shorter axis and density in the 3D map previously calculated from complexes purified in DDM [26] to anisotropy in the data set. The 3D reconstruction of 4F2hc-LAT2 presented here was calculated from a significantly larger data set, thus guaranteeing a superior uniform angular coverage (i.e., azimuthal angles) of projections (Fig. S2D) and map quality.

Purification of human membrane proteins for functional and structural studies remains a challenge nowadays. Even more challenging is the purification of covalently bound human membrane protein complexes such as heterodimers where the connecting disulphide bridge is prone to breakage. Here we significantly stabilized purified human 4F2hc-LAT2 using a combination of two detergents, i.e., DDM and LMNG, and CHS. This made possible the application of the SPA radioligand-binding assay and the reconstruction of a solid 3D map. The stabilization of the human 4F2hc-LAT2 complex paves the way towards its crystallization for structure determination by crystallographic methods, and the elucidation of the molecular working mechanism of this important protein complex.

## Materials and Methods

### Overexpression, purification and SEC of 4F2hc-LAT2

Pichia cells overexpressing human 4F2hc-LAT2 were grown in batch culture or bioreactor as described previously [25], [32]. Protein purification was performed similar to Costa *et al.* [25] using DDM (Anatrace, USA), LMNG (Anatrace, USA) and CHS (Sigma-Aldrich), and considering a 1∶5 (w/w) ratio between CHS and total detergent. Briefly, *P. pastoris* membranes containing overexpressed 4F2hc-LAT2 were solubilized for 1 h at 4°C under gentle agitation in 1.5% DDM, 0.1% LMNG and 0.32% CHS in Buffer P (20 mM Tris–HCl pH 8, 300 mM NaCl and 10% glycerol). During detergent solubilisation the protein concentration was 3 mg/ml. After ultracentrifugation (100,000g at 4°C for 1 h), 4F2hc-LAT2 was purified from the supernatant by two sequential affinity chromatography steps, i.e., cobalt (TALON, Clontech, BD Biosciences, Germany) and Strep-Tactin (Superflow, high capacity resin; IBA, Germany) affinity chromatographies. 4F2hc-LAT2 was bound to a TALON column equilibrated with Buffer P containing 5 mM imidazole, 0.2% DDM, 0.013% LMNG and 0.0426% CHS. For washing and elution, the same buffer was used containing 20 mM and 200 mM imidazole, respectively. The elution was then bound to Strep-Tactin resin and washed in Buffer P containing 0.1% DDM, 0.0065% LMNG and 0.0213% CHS. 4F2hc-LAT2 was finally eluted with the same buffer supplemented with 8 mM desthiobiotin (IBA, Germany).

SEC of purified 4F2hc-LAT2 was performed with a Superose 6 10/300 GL column (GE Healthcare) using 20 mM Tris-HCl pH 8, 150 mM NaCl, 10% glycerol, 0.1% DDM, 0.0065% LMNG, 0.0213% CHS as elution buffer. The SEC column was calibrated using the marker proteins: Conalbumin (CA, 75 kDa), Aldolase (A, 158 kDa), Ferritin (F, 440 kDa) and Thyroglobulin (TG, 669 kDa), and the molecular weight (Mw) of the complex was determined similar to Ilgü *et al.* [33].

### SPA of 4F2hc-LAT2

SPA was conducted with purified 4F2hc-LAT2 as described previously [30] using the following experimental conditions: 4.2 µg of protein, 0.5 µCi [^3^H]L-leucine (specific activity: 120 Ci/mmol, Perkin Elmer), 300 µg PVT-copper beads (Perkin Elmer) per 96-well plate (white OptiPlates, Perkin Elmer) well. For the competition (Fig. 1D, bar 2) and background control (Fig. 1D, bar 3) experiments, 4 mM L-leucine and 100 mM imidazole were also present in the mixtures, respectively. The total assay volume was always 100 µl.

### Negative-stain TEM, 3D reconstruction and on-grid Nanogold labelling

Purified human 4F2hc-LAT2 at ∼30 µg/ml protein concentration was adsorbed for ∼10 s to parlodion carbon-coated copper grids which were rendered hydrophilic by glow discharge at low pressure in air. Grids were washed with three drops of double-distilled water and stained with two drops of 0.75% uranyl formate (BDH Chemicals, USA). Electron micrographs were recorded with a Philips CM12 transmission electron microscope operated at 80 kV and equipped with a Morada CCD camera (Soft Imaging System, Olympus). For image processing and 3D reconstruction, the software package EMAN2 [34] was used. Initially, 28,993 single particles of 4F2hc-LAT2 were picked with a box size of 128 pixels using the e2boxer program. Electron micrographs were corrected for the contrast transfer function of the microscope using the e2ctf program. Reference-free classification and averaging was done with the e2refine2d program, which yielded about 200 class averages. From these class averages the e2initialmodel algorithm built a starting 3D model. The preliminary model was refined against a final set of 27,921 projections by running the iterative refinement procedure e2refine. During the refinement process the angular spacing was decreased from 15° to 2.5°. The resolution of the final 3D reconstruction was estimated by calculating the Fourier shell correlation (FSC) with the e2eotest program of EMAN2 and using the 0.5 criterion as a resolution indicator (conventional FSC calculation, not gold standard). The density threshold for volume rendering of the 4F2hc-LAT2 map was determined by fitting the surface representation of the 4F2hc ectodomain (4F2hc-ED; PDB: 2DH2) crystal structure into the small density of the 3D reconstruction and adjusting the threshold. Visualization of the 3D model and fitting of the 4F2hc-ED crystal structure was done in UCSF Chimera [35].

For Nanogold labelling, purified 4F2hc-LAT2 was adsorbed to electron microscopy grids as described above. Grids were washed with three drops of double-distilled water and blotted. Then immediately, 10 µl of 5 nm Ni-NTA Nanogold solution (Nanoprobes Inc., USA) diluted 1∶5 in 60 mM Tris-HCl pH 8, 450 mM NaCl, 2.5 mM imidazole) were added. Grids were incubated for 30 min at room temperature in a humidity chamber (Petri dish with wet paper towel inside). After incubation, grids were blotted to remove the Nanogold solution, washed with one drop of double-distilled water and finally negatively-stained with 0.75% uranyl formate.

## Supporting Information

Figure S1 **Stability of purified human 4F2hc-LAT2.** SDS-PAGE and Western blot analyses of 4F2hc-LAT2 purified in DDM, LMNG and CHS (A), and DDM only (B) after purification (day 1; *upper*) and 6 days later (*lower*). Purified protein was always kept on ice or 4°C. Western blot analysis was performed using anti-4F2hc (lanes α4F2_ED_) and anti-StrepTagII (lanes αSTag) antibodies. 10% SDS/polyacrylamide gels were used. Gels in lanes C were stained with Coomassie Blue. In (A) no disruption of the complex was observed (*upper*). Only some higher aggregates were observed 6 days after purification (*lower*). In stark contrast, 4F2hc and LAT2 monomers from disrupted heterodimers as well as some higher aggregates were found in 4F2hc-LAT2 purified in DDM after purification (B) (*upper*). Incubation at 4°C for additional 6 days dramatically increased aggregation of the complex. In all lanes C, 5 µg protein were loaded with the exception of lane C in *lower*, panel (B). Here 20 µg were loaded in order to visualize bands not corresponding to higher aggregates (which were most abundant).
(TIF)

Figure S2 **Single particle analysis and 3D reconstruction of human 4F2hc-LAT2.** (A) Representative class averages of 4F2hc-LAT2 purified in DDM, LMNG and CHS. The reference-free class averages of the heterodimer were generated from 27'921 single projections. The frame size of the class averages is 39.6 nm. (B) Overview of the 3D reconstruction of 4F2hc-LAT2. The 3D model is rotated in increments of 45° around the x- (*lower*) or y-axis (*upper*). The scale bar represents 5 nm. (C) Resolution of the 4F2hc-LAT2 3D map. According to the 0.5 criterion the Fourier Shell Correlation (FSC) function indicates a resolution of 20 Å. (D) The Euler angle distribution of single particle projections demonstrates a homogeneous sampling.
(TIF)

Figure S3 **Comparison of human 4F2hc-LAT2 3D reconstructions.** Front (A) and side (B) views of 3D maps of 4F2hc-LAT2 purified with DDM/LMNG/CHS (in grey) and with DDM only (in yellow). In (A) and (B), the X-ray crystal structure of 4F2hc-ED is shown as surface representation (PDB: 2DH2; CPK colours). In (A) the measured widths of LAT2 are different, i.e., 85 Å *versus* 71 Å, while in (B) identical, i.e., 99 Å.
(TIF)
