# Supplementary figures and images for: Detergent-Induced Stabilization and Improved 3D Map of the Human Heteromeric Amino Acid Transporter 4F2hc-LAT2
Source: PLoS One. 2014 Oct 9;9(10):e109882. doi: 10.1371/journal.pone.0109882 (PMC4192586; doi:10.1371/journal.pone.0109882)

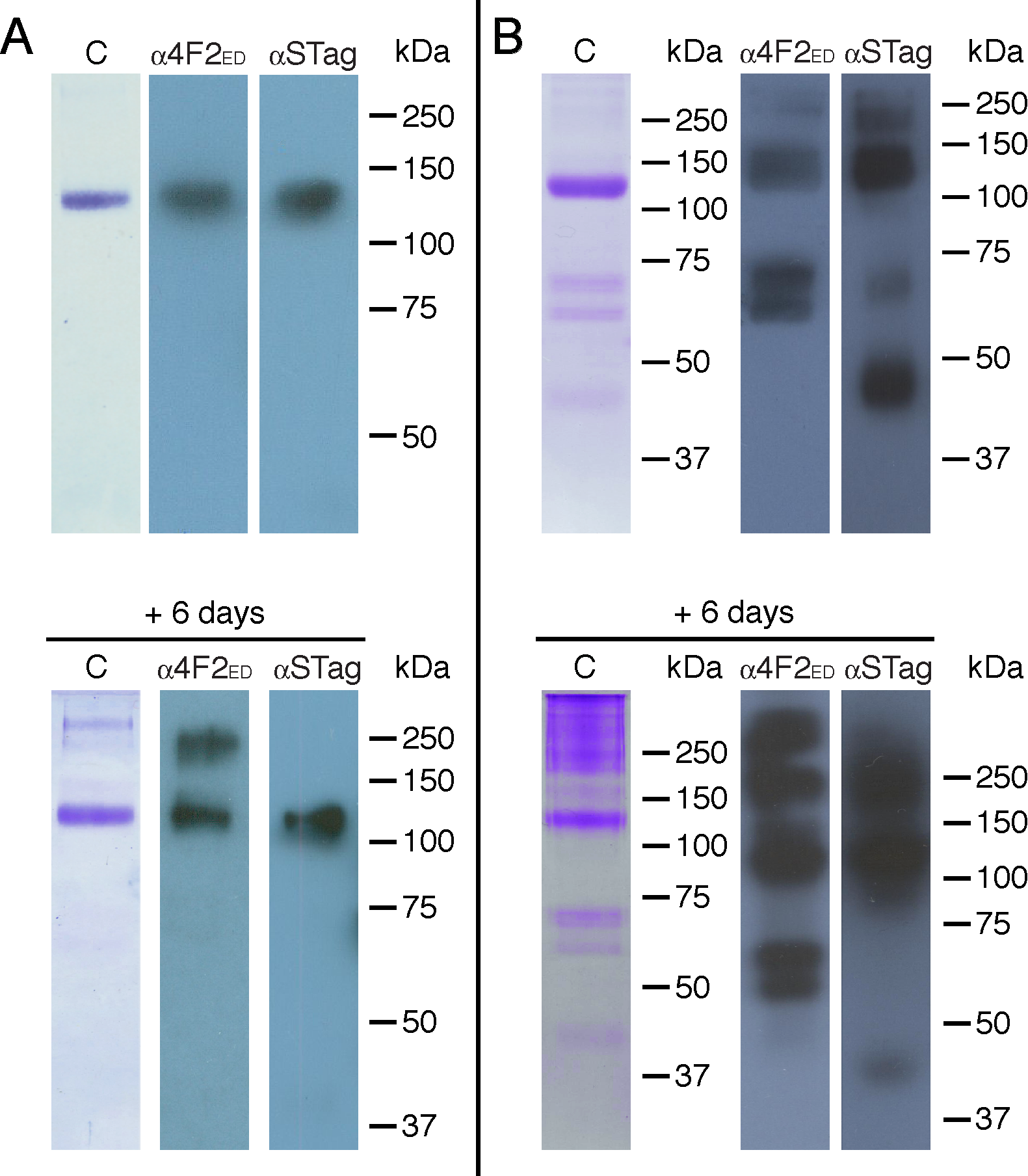

Supplement: Figure S1 — Stability of purified human 4F2hc-LAT2. SDS-PAGE and Western blot analyses of 4F2hc-LAT2 purified in DDM, LMNG and CHS (A), and DDM only (B) after purification (day 1; upper) and 6 days later (lower). Purified protein was always kept on ice or 4°C. Western blot analysis was performed using anti-4F2hc (lanes α4F2ED) and anti-StrepTagII (lanes αSTag) antibodies. 10% SDS/polyacrylamide gels were used. Gels in lanes C were stained with Coomassie Blue. In (A) no disruption of the complex was observed (upper). Only some higher aggregates were observed 6 days after purification (lower). In stark contrast, 4F2hc and LAT2 monomers from disrupted heterodimers as well as some higher aggregates were found in 4F2hc-LAT2 purified in DDM after purification (B) (upper). Incubation at 4°C for additional 6 days dramatically increased aggregation of the complex. In all lanes C, 5 µg protein were loaded with the exception of lane C in lower, panel (B). Here 20 µg were loaded in order to visualize bands not corresponding to higher aggregates (which were most abundant). (TIF) [file pone.0109882.s001.tif]

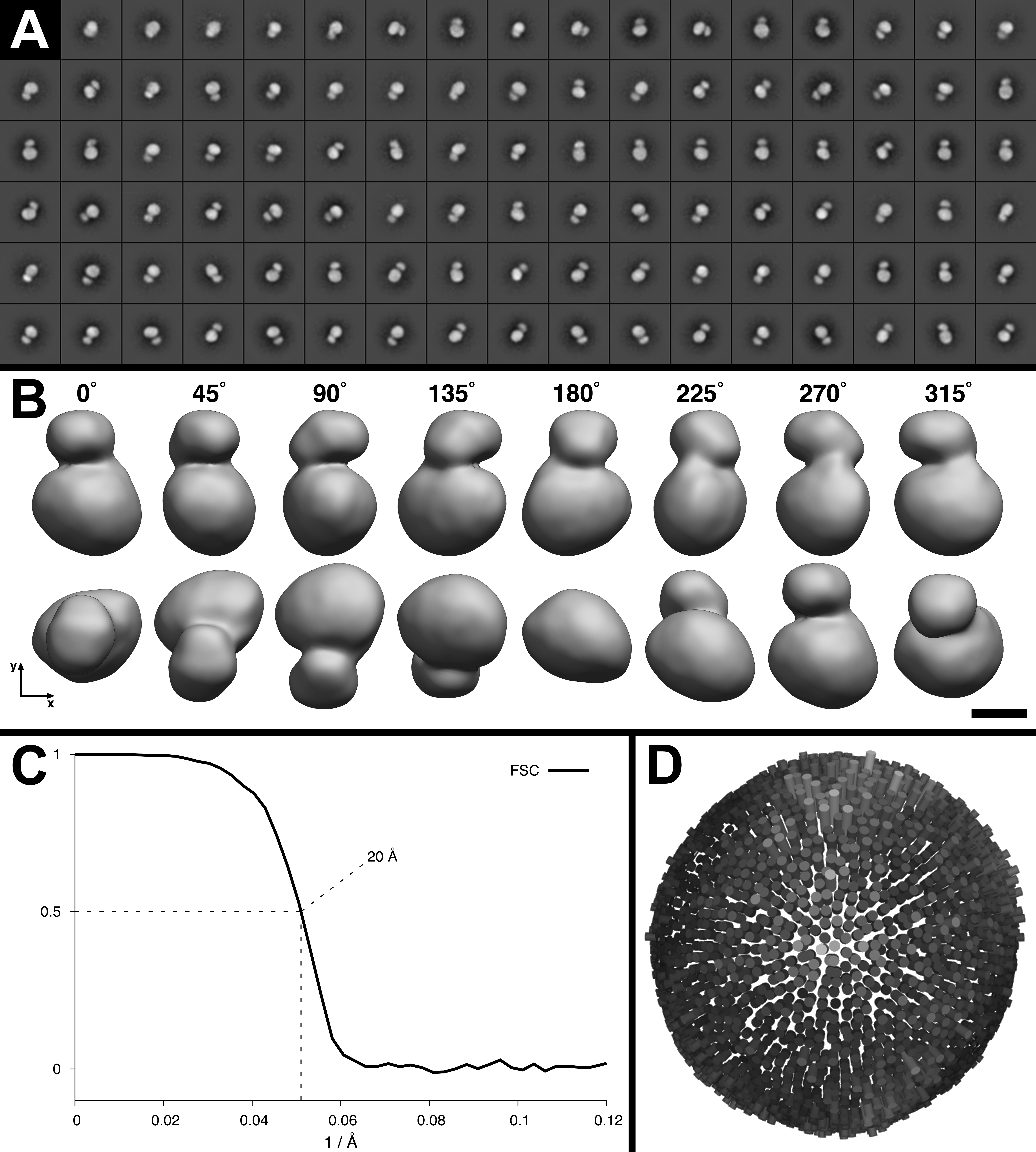

Supplement: Figure S2 — Single particle analysis and 3D reconstruction of human 4F2hc-LAT2. (A) Representative class averages of 4F2hc-LAT2 purified in DDM, LMNG and CHS. The reference-free class averages of the heterodimer were generated from 27'921 single projections. The frame size of the class averages is 39.6 nm. (B) Overview of the 3D reconstruction of 4F2hc-LAT2. The 3D model is rotated in increments of 45° around the x- (lower) or y-axis (upper). The scale bar represents 5 nm. (C) Resolution of the 4F2hc-LAT2 3D map. According to the 0.5 criterion the Fourier Shell Correlation (FSC) function indicates a resolution of 20 Å. (D) The Euler angle distribution of single particle projections demonstrates a homogeneous sampling. (TIF) [file pone.0109882.s002.tif]

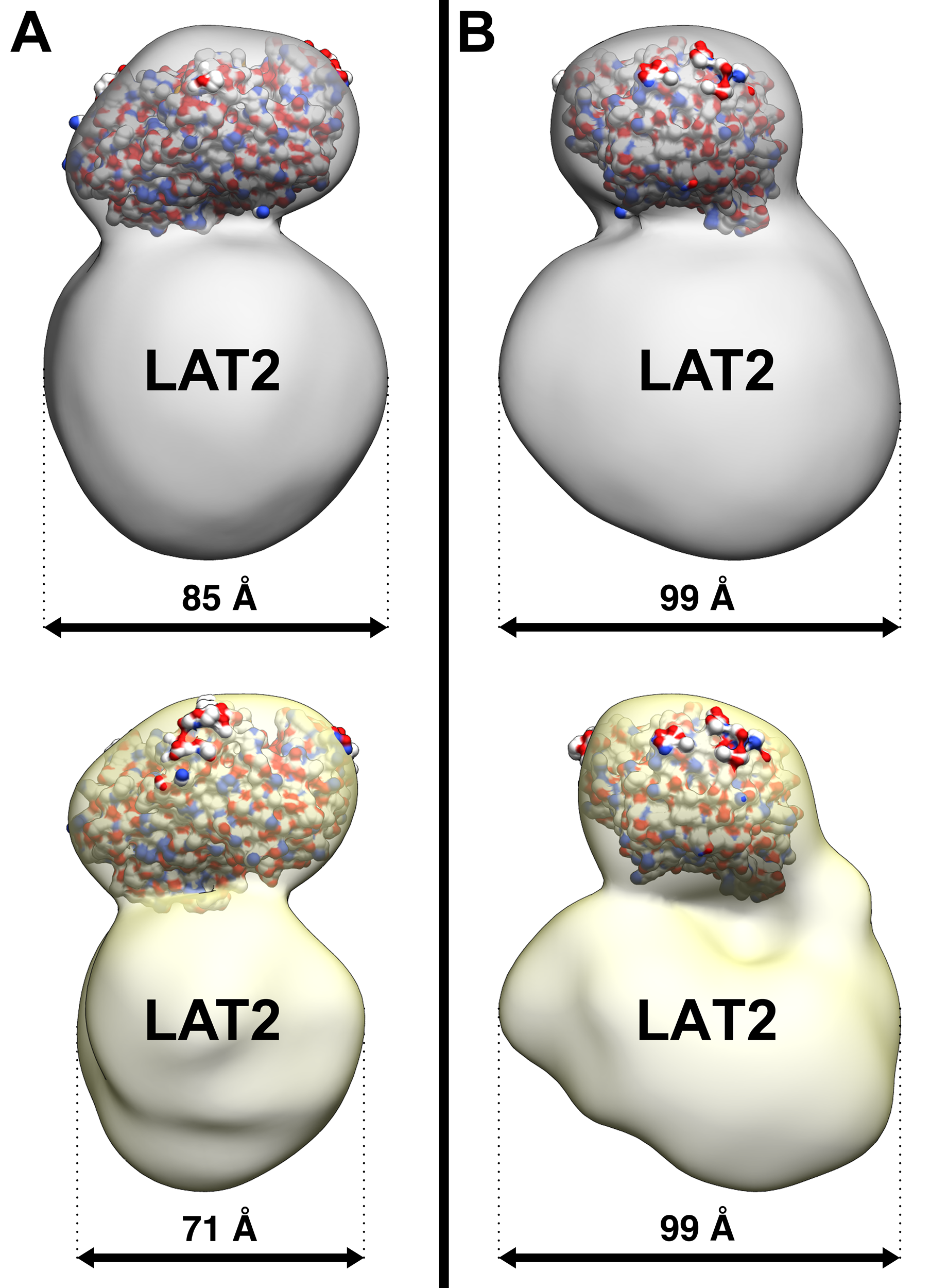

Supplement: Figure S3 — Comparison of human 4F2hc-LAT2 3D reconstructions. Front (A) and side (B) views of 3D maps of 4F2hc-LAT2 purified with DDM/LMNG/CHS (in grey) and with DDM only (in yellow). In (A) and (B), the X-ray crystal structure of 4F2hc-ED is shown as surface representation (PDB: 2DH2; CPK colours). In (A) the measured widths of LAT2 are different, i.e., 85 Å versus 71 Å, while in (B) identical, i.e., 99 Å. (TIF) [file pone.0109882.s003.tif]
